# The Post-Antibiotic Era: Promising Developments in the Therapy of Infectious Diseases

**Published:** 2010-06

**Authors:** Mario Zucca, Dianella Savoia

**Affiliations:** *Department of Clinical and Biological Sciences, Faculty of Medicine S. Luigi Gonzaga, University of Torino, Torino, Italy*

**Keywords:** antibiotics, antibodies, antimicrobial peptides, antivirulence factors, bacteriophages, quorum sensing

## Abstract

An overview of investigational antibiotics highlights that antimicrobial drug development is slower than the emergence and spread of resistant strains. In the last three decades only two antibiotics belonging to truly new classes have been introduced into the market, i.e. linezolid and daptomycin. This situation is fostering a huge amount of research aimed at the development of novel molecules and novel antibacterial approaches. The present review details the state of the art research in the fields of antimicrobial peptides, antivirulence factors, bacteriophages, and antibodies as possible replacements or enhancers of classic antibiotics. If the number of new antibacterials in phase II or III of clinical trials remains disappointing, it seems nonetheless reasonable to expect major breakthroughs, made possible by the synergistic use of computational methods and chemical and biological research.

## INTRODUCTION

After 60 years of use, antibiotics are progressively decreasing their efficacy against infections. Extremely multi-resistant bacteria, such as methicillin-resistant *Staphylococcus aureus* (MRSA), vancomycin-resistant *Enterococcus faecium*, and fluoroquinolone-resistant *Pseudomonas aeruginosa*, take a consistent death toll. According to the Infectious Diseases Society of America, it is estimated that in US hospitals more people die of MRSA infection than of AIDS and tuberculosis combined (1). The emergence of resistant bacteria is generated by the strong evolutionary pressure inherent to the mechanism of action of antibiotics and to their increasingly wide-spread use. On the other hand, the cost and the difficulty to identify, characterize and licence new antibiotics has progressively and dramatically lowered the number of new antibacterial agents approved for marketing. In the last 30 years only two antibiotics with a novel mechanism of action, both active against Gram-positive cocci, have been introduced into the market: linezolid, a oxazolidinone that inhibits protein synthesis, and daptomycin, a cyclic lipopeptide that depolarizes cytoplasmic membranes (2). New hope comes from the discovery that a huge range of natural products may be developed as novel antimicrobial agents. After the discovery of the antibacterial activity of polymixins, basic and applied research is extensively investigating the field of antimicrobial peptides (AMPs), that are produced by species of all kingdoms, ranging from bacteria, fungi and plants to mammals and non-mammalian vertebrates. Hundreds of these peptides possess direct antimicrobial activity against bacteria, fungi, eukaryotic parasites and/or viruses, and some of them are being developed to obtain more active synthetic or semi-synthetic analogues (3). In recent years the complete sequencing of many bacterial genomes and the availability of new molecular biology techniques such as proteomics, genomics and RNA inhibition led to new insights on host-pathogen interactions. Progressive identification and thorough characterization of many bacterial functions that concur to the virulence of a given bacterial strain make the possibility to target virulence factors quite real and very attractive. The search for anti-virulence factors, including synthetic substances and many natural products derived from animal, vegetal, mycological and bacterial sources, is a rapidly growing field. Other promises come from the possibility to use natural or engineered antibodies as antimicrobial tools, and from bacteriophages, that are already currently used worldwide to control bacterial growth in the food industry. Their medical use in the treatment of bacterial infections, by now limited to Eastern Europe and to the former Soviet Union, is earning a renewed interest in Western countries (4).

The scope of this brief review is to provide a survey of the most recent work that is being performed to identify and bring into clinical use antibacterial molecules that differ from classic antibiotics for their innovative mechanism of action. The paper deals with the recent insights on antimicrobial peptides, antivirulence factors, antibodies and bacteriophages, focusing on the mechanisms of these new strategies but avoiding extensive molecule lists, that anyway would become obsolete in the next few months. Considering the ever-increasing technical breakthroughs, it can be envisaged that in the next two decades some molecules belonging to the above indicated categories will become useful therapeutic tools.

## ANTIMICROBIAL PEPTIDES

The concept of using AMPs as therapeutic tools was first introduced in the late 1990s, however none of them has yet reached the market (5). AMPs are evolutionary conserved factors (6) with pleiotropic activities and intriguing potential: they participate to the innate immune response representing the first line of defence against pathogens, and combine antimicrobial activity with angiogenic, immunomodulating and anti-inflammatory properties (7). Based on their electrical charge, AMPs can be divided into anionic peptides rich in glutamic and aspartic acid (maximins H5 from amphibians, dermicidins from humans), and into the most thoroughly studied cationic peptides (CAMPs). According to their molecular and conformational structure, in turn CAMPs can be divided into four classes: cysteine-rich β-sheet structures with one or more disulphide bonds (defensins from humans); linear α-helical peptides without disulphide bonds (cecropins, magainin, dermaseptin from amphibians, LL37 from humans); loop-structured peptides (microcins from *Enterobacteriaceae*) (8), and extended tryptophan-rich peptides (cathelicins, indolicidin) (9). Another AMP class includes peptidoglycan recognition proteins (PGRPs), that have been identified at first in the silkworm (10) and subsequently in insects, humans ad mice (11), but are not currently being developed as antibacterial drugs. A broad review of the fifteen hundred AMPs identified over the last 20 years is beyond the scope of this review: we will focus on some of them and on their possible use in antimicrobial therapy. The exact mechanism of action of CAMPs has yet to be established, but according to the most accepted model the initial phase is common and consists in an electrostatic interaction with the target cell. Subsequently, two kinds of mechanisms have been defined: the rapid disorganization of the cytoplasmic membrane, that takes seconds to minutes, and the binding of the molecules to intracellular targets, that takes more time (3-5 hours).

Defensins, produced by vertebrates, plants and insects, are active against a broad spectrum of bacteria, fungi, protozoa and enveloped viruses (12). The development of human defensins for therapeutic use has been hindered by different and as yet unsolved problems, such as the technical challenge of producing them at the scale and purity required for a pharmaceutical product, their ability to stimulate the immune system, and their direct toxicity on mammalian cell membranes (12). However plectasin, a defensin produced by the fungus *Pseudoplectania nigrella*, shows good activity against a broad spectrum of Gram-positive bacteria, and low cytotoxicity against mammalian cells. The molecule, whose mechanism of action involves the binding to the bacterial cell wall precursor lipid II, is currently under development by Novozymes A/S (13).

Cecropins and magainins kill microorganisms by membrane permeabilization, with a detergent-like effect accompanied by pore formation. (14-16). This mechanism is quite rapid, concentration dependent and, most important, it does not need the interaction with a specific receptor, thus avoiding the induction of resistance. CAMP specificity of action depends upon the differences in the composition and physicochemical properties of germ and host cell membranes. For example, magainin induces pore formation in bacterial membranes rich in anionic phospholipids, but not in animal cell membranes, rich in neutral phospholipids and further stabilized by cholesterol.

Microcins are antibacterial peptides, mainly secreted by *E. coli* species, involved in the regulation of microbial competition within the intestinal microbiota. These molecules exert potent antibacterial activity being active in the nanomolar range, and have a peculiar mechanism of action defined as a “Trojan horse” behaviour: they are recognized as siderophores by outer membrane receptors of susceptible bacteria and internalized; once inside the cell they bind essential enzymes or interact with the inner membrane killing the bacterium (17).

Originally isolated from bovine neutrophils, indolicidin is a CAMP rich in tryptophan and proline residues. It is believed that indolicidin performs its action reaching bacterial cytoplasm, where its amphipathicity allows its interaction with other proteins, and its cationic surface allows its interaction with both the negatively charged bacterial membranes and the negatively charged phosphate backbone of DNA (18). Indolicidin is a highly potent antibacterial, but its cytotoxicity barred its therapeutic use. However, less toxic novel derivatives showing promising pharmaceutical potential are currently under development. Omiganan, a synthetic indolicidin homologue, has demonstrated *in vitro* activity against a wide range of Gram-positive and Gram-negative bacteria and fungi. It is now under clinical development for the prevention of catheter-related infections and for the treatment of acne and rosacea (9).

Mechanisms underlying the specificity of action against Gram-positive or Gram-negative bacteria are still poorly understood. It seems that the killing of Gram-negative bacteria relies upon the ability to cross their external membrane. Some CAMPs interact with membrane or cytoplasmatic receptors targeting intracellular proteins, such as heat shock proteins (19, 20). A number of CAMPs have been tested or developed for clinical use, especially for topic treatments (21). The first that has reached the phase III trials is Pexiganan, a magainin II homologue. It was evaluated as an antibiotic cream for foot ulcers, but it was refused a license by the U.S. FDA in 1999 for questionable efficacy (9). Iseganan, a protegrin I homologue, was tested against oral mucositis, but also failed the efficacy test. Bloodstream infections arising from multidrug-resistant strains are an increasingly alarming threat, especially in immunocompromised patients. Four promising peptides, available for i.v. administration only, are currently under investigation: dalbavancin, a novel semisynthetic lipoglycopeptide that inhibits cell wall synthesis and is especially active against MRSA, is undergoing phase III clinical trials for skin and soft tissue infections and catheter-related bloodstream infections (22); telavancin and oritavancin, that share their mechanism of action with dalbavancin; human lactoferrin 1-11, that is being tested in patients with *Staphylococcus epidermidis* bacteremia and in patients with candidemia (23). To date, most clinical trials have focused on the topical use of peptides, as the oral and i.v. administration routes pose two orders of challenges: the limited stability of the molecules inside the host, where they are exposed to degradation by intestinal, tissue and serum proteases, and the still unknown toxicology. Possible side effects could manifest as direct and immediate cellular damage or as a delayed effect on the immune response. These issues, currently under extensive investigation, are the main causes of the delay of the AMP availability for clinical use.

## ANTIVIRULENCE FACTORS

Most traditional antibiotics target processes essential for *in vitro* growth, with the implicit assumption that the same processes are essential for *in vivo* growth also. However, recent work performed on fatty acid biosynthesis inhibitors evidences that in some cases there is a potential disparity between requirements for *in vivo* and *in vitro* bacterial survival (24). Bacterial functions that cause disease *in vivo* usually fall into two categories: those required for *in vivo* survival, that in some cases may be also essential for *in vitro* survival, and those that cause tissue damage and disease. The latter, together with factors that interfere with host immune functions, are classically considered virulence factors.

Virulence factor targeting compounds have at least two advantages over classic antibiotics: firstly, they do not exert a selective pressure on bacteria, as they do not target genes essential for bacterial viability *in vitro*, but only affect functions essential for host/pathogen interaction; secondly, the specificity of the effect should preserve the normal flora constituted by non-virulent resident bacteria (25). Another possible advantage is that the host, being exposed to intact but harmless bacteria, will develop a sound immune response against a possible reinfection by the virulent form (26). The relevance of the approach based on virulence factor targeting is underscored by the remarkable amount of correlated research, that is producing the first practical results. Recently, Nielsen *et al*. have developed a new simple assay to screen compounds that affect *Staphylococcus aureus* virulence gene expression (27). On the other hand, the identification of antivirulence molecules poses two orders of challenges: i) to identify targets that are constitutively expressed by bacteria, and ii) the availability of standardized functional assays that should mimic the *in vivo* conditions of the infectious process, considering that by definition virulence inhibitors will not kill bacteria *in vitro* (24). Moreover, the clinical use of such drugs would rely on rapid diagnostic methods, because the specificity of action is linked to a limited spectrum of activity. However, the recent availability of many thoroughly sequenced bacterial genomes makes it possible the selection of antivirulence factors active against conserved targets common to different bacterial species.

The two main early steps in the establishment of an infection involve pathogen adhesion to the host and the successive pathogen replication, i.e. the host colonization. Bacterial adhesion has been thoroughly studied in relation to *E. coli* urinary tract infections. It has been observed that the prevalence and degree of bacterial adherence to uroepithelial cells are closely associated with the clinical category of urinary tract infections. Of isolates from patients with pyelonephritis, or bacteremia, 70 to 100% adhere to uroepithelial cells (with a mean of approximately 30 bacteria per cell), compared with 50 to 60% of strains (with about 20 bacteria per cell) among cystitis patient isolates, 22 to 36% of strains (with about 10 bacteria per cell) among asymptomatic bacteriuria patient isolates, and 10 to 36% of fecal strains (with about seven bacteria per cell) (28). Adhesion and colonization are the result of a complicate interplay between the germ virulence factors and the early immune mechanisms of the hosts, and their neutralization should allow the early interruption of the infectious process.

A different antivirulence approach consists in the inhibition of specific bacterial toxins, that mediate the bulk of pathology observed in several types of bacterial infections.

### Inhibitors of adhesion

Bacterial adhesion to host cells can be mediated by protein molecules present on the surface of the bacterium or by proteinaceous short filaments called pili or fimbriae protruding from the bacterial surface. Gram-positive bacteria such as *Staphylococci* possess many different cell-wall anchored proteins that play key roles in the infection process, as they mediate bacterial binding to the host cells, acquire essential nutrients, and circumvent the immune respose (29). The mechanism involved in the anchoring of these proteins to the cell-wall is conserved in almost the entire class of Gram-positive bacteria, and is mediated by a class of cysteine transpeptidases called sortases, that are by now considered an attractive potential target for specific inhibitors. A number of different strategies, such as screening natural products and small compounds libraries, or synthesizing rationally designed peptidomimetics, have been employed to search for sortase inhibitors. Recently Suree *et al*. have identified two promising small molecules that inhibit *S. aureus* SrtA sortase but do not impair bacterial growth *in vitro* (30).

Pili, that are considered the major mediators of bacterial adhesion, are produced by many Gram-negative bacteria, including *Neisseriae* and fermenting and non-fermenting rods. Genome sequencing of meningococci led to the discovery of several other adhesins, which are normally expressed at low levels *in vitro* and could be up-regulated *in vivo*, but their potential roles in pathogenesis remain to be fully defined (31). In this section we will now deal more in detail with *E. coli* pili and their inhibitors, because this system is the most investigated and the one that shows the possibility of most promising developments in the immediate future. *E. coli* adhesion can be prevented by the inhibition of pili formation or of pili binding to the surface of mammalian cells. Pili are multi-protein fibers, that are assembled in the periplasmic space via a highly conserved mechanism called the chaperone-usher pathway, common to different Gram-negative species such as *E. coli, P. aeruginosa, Yersinia enterocolitica, Haemophilus influenzae, and Bordetella pertussis* (26). Beyond type 1 pili, uropathogenic *E.coli* (UPEC) produce type P pili, more frequent in pyelonephritis-associated strains, and curli, i.e. extracellular amyloid fibers that are major components of the bacterial extracellular matrix (32). It has been demonstrated that pili- and curli-expressing *E. coli* adhere better to uroepithelial cells than non-piliated or non-curliated strains do (33). Small synthetic compounds of the family of N-substituted amino acid derivatives and substituted bicyclic 2-pyridones, called pilicides, have been developed on the basis of the known molecular details of the interaction of pili subunits with the chaperone proteins (26). By competitively inhibiting the chaperone protein function, pilicides dose-dependently decreased type 1 pili production by UPEC. Pilicides share a common chemical lineage with FN075 and BibC6, two ring-fused 2-pyridones that inhibit curli biogenesis by preventing the polymerization of the major curli subunit protein CsgA (34). Biological evaluation showed that the reduction of pili consistently correlated with the loss of UPEC’s ability to colonize bladder cells and to form biofilms (26). Curli reduction significantly attenuated UPEC virulence in a murine model of urinary tract infection (34). Another possibility to inhibit fimbriated *E. coli* adhesion to cells involves the use of a new family of well defined small macromolecules, called glycodendrimers. These molecules mimic glicans present on the surface of mammalian cells and prevent adhesion by binding to pili (35). The potential clinical applications of these approaches are currently investigated, and these studies can be considered a first step in the development of new therapeutic tools.

### Inhibitors of colonization

In many instances the successful establishment of an infection depends upon the ability of the involved bacteria to form biofilms, i.e. large colonies of bacteria that adhere to biotic or abiotic surfaces and behave as an organized community reacting to small diffusible signal molecules termed autoinducers or quorum sensors (36). Gram-negative bacteria mainly produce acyl-homoserine lactone (AHL) autoinducers, whereas Gram-positive bacteria use a two-component sensory system and oligopeptide autoinducers (37). Quorum sensors are constitutionally produced and secreted by bacteria, and when their concentration reaches a threshold value that depends upon bacterial population density (quorum), they interact with species-specific receptors belonging to the LuxR family of response regulators. LuxR homologues contain two domains, an AHL-binding domain and a DNA-binding domain, that upon ligand binding acts as a transcriptional activator. The whole system is termed quorum sensing (QS).

QS systems generally offer three points of attack: the signal generation, the signal molecule, and the signal receptor. *In vitro* AHL production blockage has been achieved by the use of substrate analogues, such as L/D-S-adenosylhomocysteine, sinefungin and butyryl-S-adenosylmethionine (38), but none of them have been tested on bacteria *in vivo*. Halogenated furanones C30 and C56 can reduce bacterial adherence to catheters and increase *P. aeruginosa* clearance in a murine model of pneumonia (37). In 2008 Skindersoe *et al*. performed a screening of 12 antibiotics for their inhibitory activity on *P. aeruginosa* QS. Their results indicate that aztreonam (AZM), ceftazidime and ciprofloxacin are very active in decreasing the expression of QS-regulated virulence factors by altering the cell membrane permeability (39). *In vitro* AZM at 2 mcg/ml down-regulated the expression of 277 *P. aeruginosa* genes and reduced AHL production by more than 70%.

The second possible strategy, i.e. QS signal inactivation, can be achieved by chemical or enzymatic degradation of the molecule, or by its antibody-mediated functional inactivation. A simple way to inactivate AHL is to break the lactone ring by increasing the pH to above 7. This strategy is adopted by a number of plants against invading bacteria. Enzymatic lactonolysis is instead operated by bacteria of the genus *Bacillus*, and by *Arthrobacter sp., Klebsiella pneumoniae, Agrobacterium tumefaciens*, and *Rhodococcus* sp. It seems that the production of AHL-degrading enzymes is employed by these bacteria in the competition with AHL producers (40). In theory, these enzymes could be used in therapy, but the drawback is that the lactonolysis reaction is reversible at acidic pH, regardless of the method used to open the lactone ring, and the pH of the milieu in inflammation sites tends to be acid. However, the lactone ring is not the only site of the AHL molecule that can be chemically targeted. It has been shown that oxidized halogen compounds, such as hypobromous and hypochlorous acids, interfere with QS-controlled gene expression. The possibility to inactivate the QS signal by means of antibodies has been explored by Miyairi *et al*. (41). These Authors observed that mice immunized by subcutaneous injection of 3-oxo-C_12_-HSL-BSA conjugate produced significant amounts of specific antibody in serum and showed a significant increase of survival upon intrapulmonary challenge with a *P. aeruginosa* suspension.

The third and most investigated strategy to interfere with bacterial QS is the classical pharmacological approach to receptor antagonism. Historically, the first examples of QS-blocking compounds were the halogenated furanones produced by the australian microalga *Delisea pulchra* (42). In order to obtain more active compounds, a screening of synthetic analogues and a structure-function analysis were performed by different groups (43). The results showed that C-30 and C-56 furanones were the most effective in reducing QS-related gene expression of *P. aeruginosa*, and in a mouse model of pulmonary infection both compounds induced a significantly faster clearance of bacteria (44, 45). Another extensively explored possibility is to displace AHL signal molecules with inactive AHL analogues obtained by chemical modification of the acyl side chain of the molecule, or of the lactone ring, or of both. It has been observed that the modification of the length of the acyl chain does not affect AHL activity, but results in the production of AHL competitive agonists. However, competitive AHL analogues have been obtained by replacing the C-3 atom with sulphur in the acyl side chain, or by placing aryl substituents at the end of the side chain (46). Molecules more chemically unrelated to AHL, such as 3-oxo-C12-(aminocyclohexanone) also possess QS inhibiting activity. Several QS inhibitors unrelated to the signal molecules have been identified screening random compound libraries (47). Among these, 4-nitro-pyridine-*N*-oxide (4-NPO) significantly lowers the expression of 37% of the QS-regulated genes in *P. aeruginosa*. Beyond the above cited halogenated furanone from *Delisea pulchra*, a number of plant extracts and natural compounds inhibiting *P. aeruginosa* QS has been identified by Rasmussen *et al* (48, 49): bean sprout, chamomile, carrot, garlic, habanero (*Capsicum chinensis*), propolis, water lily, yellow pepper, and two products of *Penicillium* fungi, patulin and penicillic acid. These Authors further investigated the effects of garlic extract, which contains at least three different QS inhibitors and was able to inhibit QS in a concentration-dependent manner. GeneChip analysis revealed that garlic extract had a profound effect on QS-regulated virulence genes, significantly reduced *P. aeruginosa* biofilm tolerance to tobramycin, and lowered the pathogenicity of *P. aeruginosa* in a *Caenorhabditis elegans* nematode model. The phytoalexin resveratrol (3,5,4’-trihydroxystilbene), an antifungal agent found in grapes and other plants, shows direct antibacterial activity against *Neisseria gonorrhoeae* and *N. meningitidis*, but not against *P. aeruginosa* (50). However, we observed that resveratrol can inhibit *P. aeruginosa* QS *in vitro* (51). Finally, solenopsin A, a venom alkaloid from the fire ant *Solenopsis invicta*, has been shown to be able to interfere with *P. aeruginosa* QS (52).

### Inhibitors of toxin production and secretion

In many instances toxins have a direct role in causing diseases. Therefore, toxin transcription, expression, and function would make excellent targets for novel antimicrobials. The efficacy of the inhibition of toxin transcription has been demonstrated in a murine model of cholera infection by Hung *et al*. (53). These Authors observed that virstatin, an inhibitor of cholera toxin and toxin-coregulated pilus expression, blocked intestinal colonization with *Vibrio cholerae*. Another example is the efficacy of a synthetic methionyl-tRNA inhibitor, that improved survival in a hamster model of *Clostridium difficile* infection (54). The potential use of *Bacillus anthracis* as a bioweapon by terrorists has prompted the research of anthrax toxin inhibitors. A secreted zinc-dependent metalloprotease, known as lethal factor (LF) because it directly kill host cells, is the major toxin involved in anthrax pathogenesis. A hydroxamate synthesized at Merck Research Laboratories can inhibit LF protease activity *in vitro*, and protected mice from a challenge with lethal doses of LF or *B. anthracis* spores (55). This molecule has been further modified and extensively tested in pharmacological and animal model studies. Several other groups have successfully developed potent LF inhibitors, that are currently under development but none has yet the clinical trial step (56).

## ANTIBODIES

Antibodies (Abs) are emerging as an important class of therapeutic factors in the field of oncology and infectious diseases (57). The potential of Abs targeting virulence factors has been shown in animal models of lung infection with *P. aeruginosa* (58). The type III secretion system is a virulence mechanism associated with severe disease and increased mortality in patients with ventilator-associated pneumonia caused by *P. aeruginosa*. The system consists of three separate protein complexes: the secretion apparatus, the translocation or targeting apparatus (PcrV protein), and the secreted toxins with their cognate chaperons. Following positive results obtained in *in vitro* and *in vivo* murine models, the efficacy of monoclonal Abs against PcrV is currently under investigation in a phase I/II human trial (59). The importance of Abs as direct effectors in bacterial killing has been pointed out by studies on the organisms causing Lyme disease and relapsing fever, i.e. *Borrelia burgdorferi* and *B. recurrentis*, respectively. Bacteria present in the bloodstream are not affected by complement, because by binding Factor H and C4BP they inhibit both the alternative and the classical complement pathway (60). However, Abs are required for recovery in both diseases. Indeed, there are numerous Abs that can kill bacteria in a complement-independent manner. The finding that monovalent Fab fragments exert bactericidal effects suggests that agglutination is not involved (60). The bactericidal function resides in the variable region, that can eliminate the entire serotype population to which it is specific (61). The direct effect of the antibody on the outer membrane of *Borrelia*, resulting in the osmotic lysis of the membrane and in bacterial death, mimics the action of many antimicrobial peptides.

A different and more sophisticated approach exploits the possibility to produce anti-idiotypic Abs that mimic the effect of AMP. A series of polyclonal and monoclonal recombinant single-chain microbicidal Abs have been produced by idiotypic vaccination with a monoclonal antibody that neutralizes a killer toxin (KT) obtained from *Pichia anomala* (62). These anti-idyotipic Abs showed potent *in vitro* microbicidal activity against pathogenic eukaryotic and prokaryotic microorganisms, such as *Candida albicans, Pneumocystis jiroveci, Leishmania major* and *infantum, Mycobacterium tuberculosis* and Gram-positive cocci (63). Systemic treatments with Abs are however limited by inherent Abs antigenicity. To overcome this problem, taking advantage of the Abs modular domain architecture, alternative reduced formats have been generated, mostly devoid of the Fc region. The study of Abs mimicking the wide-spectrum antimicrobial activity of KT allowed the synthesis of peptides with antifungal, antiviral and antitumor activity (57).

## PHAGE THERAPY

The first paper concerning bacteriophages was published in 1896, when Hankin reported that a filterable factor present in the waters of the Ganges and Jumna rivers had marked antibacterial activity (64). About 20 years later Frederick Twort isolated filterable entities capable of destroying bacterial cultures (65). Twort did not further explore his findings, but two years later Felix d’Herelle, working at the Pasteur Institute in Paris, reported the same phenomenon. d’Herelle identified the new factor as a virus parasitic on bacteria, called it “bacteriophage” and promoted its use in the treatment of bacterial infections (66). Since then, several studies demonstrated that bacteriophages can be successfully used in the therapy of animal and human bacterial infections (67), but their human use in Western countries has been hindered so far by cost, safety concerns about injecting phages in the bloodstream, and by the inconsistent outcome of the treatments, often due to the poor characterization of bacteriophage preparations. To be used in therapy to their full potential, phages should be free of direct adverse side effects on the host, should not carry virulence factors or transfer antibiotic resistance genes among bacteria, and should be able to target pathogens but spare commensal bacteria. The state of the art of phage product development has been recently reviewed by Housby and Mann (68). Phage therapy is already applied in the agricultural, food-processing and fishery industries, and is used for the treatment of bacterial infections in Georgia and Eastern Europe, but there is a need for further carefully controlled empirical data on its efficacy and safety (69).

The most obvious use of bacteriophages consists in the exploitation of their ability to infect and kill susceptible bacteria. This approach has the advantage of the self-amplification of the therapeutic factor at the infection site, that can overcame bacterial defence mechanisms like biofilm formation, but the success of the treatment depends upon many variables that may affect every single step of the lytic bacteriophage replication cycle. The *in vivo* pharmacokinetics of phages are highly complex (70), being influenced by the clearance rate of the phages by the immune system of the host and by the fact that bacteria may become phage-resistant by lysogeny or by mutations concerning metabolic steps or surface receptors. Recent experiments performed by Fu *et al*. (71) on the efficacy of a bacteriophage cocktail to prevent the formation of *P. aeruginosa* biofilms on catheters in an *in vitro* model suggest the potential of the approach, that reduced the number of bacteria by 99.9%, but did not succeed in completely eradicating the bacteria. In our view, the real weakness of this approach resides in the fact that, as is the case with auto-vaccines, to be effective the therapeutic phage should be grown on bacteria isolated from every single patient. This means that results would be quite irreproducible, and, more important, that the process does not fit with the production philosophy of the pharmaceutical industry.

A different approach that can overcome these problems involves the use of purified bacteriophage products as anti-infective agents. Bacteriophage endolysins are mureine-degrading enzymes, originally studied and developed to control mucous membrane infections (72). They work on Gram-positive bacteria only, and have been found active against *Bacillus anthracis* (73), *Streptococcus pneumoniae* (74) and *Streptococcus agalactiae* (75), alone or in combination with conventional antibiotics or lysozyme. Lysins have a short half-life (15-20 min), but their action is so rapid that nanogram quantities of specific lysin can kill specific Gram-positive bacteria in seconds after contact (76, 77). Experiments performed on murine models showed that endolysins are *per se* free of toxicity and, unexpectedly, are not easily inactivated by antibodies (78). The possible toxic effect of the release of proinflammatory molecules by lysed bacteria has also been addressed. Endotoxin, teichoic and lipoteichoic acids, and peptidoglycan massive release could result in septic shock and multiple organ failure, but so far no side-effects related to lysine-induced bacteriolysis have been reported (79). According to experiments performed on a murine model, lysins may be able to cure already established infections (80). Basic applications of endolysins include the elimination of bacteria from mucous membranes, the treatment of bacterial infections, and the biocontrol of bacteria in food. Based on these results and considering that the endolysin target, peptidoglycan, is not present in eukaryotic cells, it can be anticipated that they will also be well tolerated by humans.

Phage tail-like bacteriocins, high-molecular-weight molecules produced by a number of Gram-negative bacteria (81), work by forming pores in the bacterial cell wall, leading to ion loss. Due to their molecular size, bacteriocins are inactive against intracellular bacteria (82). In recent years a number of studies report experiments performed *in vivo* on the activity of bacteriophages or their products against *Escherichia coli, Enterococcus fecalis, Staphylococcus aureus, Klebsiella pneumoniae* (83), *Pseudomonas aeruginosa* (84), and *Yersinia enterocolitica* (80). In some cases, bacteria produce and secrete phage-like molecules to obtain competitive advantages. R-type pyocins are high-molecular-weight bacteriocins carried within the chromosomes of some bacterial species, such as *P. aeruginosa*, and almost certainly evolved from lysogenic bacteriophages of the *Myoviridae* family. They bind with their tail fibers to bacterial surface and kill by inserting a core or needle that alters the bacterial membrane. Their mechanism of action, high bactericidal potency (one pyocin particle can kill one bacterium), and focused spectrum suggest that R-type pyocins could be developed as antibacterial agents (85).

## CONCLUSIONS

Despite the huge amount of basic research currently underway, the number of antibacterials in phase II or III of clinical development remains disappointing. In the last decade the concept of antivirulence factors as a new generation of antibacterial agents became an established theory, and an increasing number of examples of successful applications of such strategy in *in vivo* animal infection models support the concept in reality (24). Phage therapy still needs crucial breakthroughs to be accepted in Western countries, whereas AMP and Abs may be considered prototypes of innovative engineered drugs.

Several compounds in early development appear promising, but phase II clinical studies are not yet underway (1). It is possible that over the next 5-10 years the number of approved antibacterials will be similar to that of the past 5 years (about 1 drug per year). However, we can envisage that peptide-based drugs represent an excellent starting material for drug discovery, due to their chemical, physical and conformational diversity (86). If we consider the huge amount of challenging biological problems that in nature are solved by peptides, and the recent scientific and methodological advances, it seems reasonable to expect major breakthroughs made possible by the synergistic use of computational methods and chemical and biological research (86). Shape complementarity is a critically important factor in molecular recognition among drugs and their biological receptors. The notion that molecules with similar 3D shapes tend to have similar biological activity has been recognized and implemented in computational drug discovery tools for decades. But the low computational efficiency and the lack of widely accessible software tools limited the use of early shape-matching algorithms. However, recent development of fast and accurate shape comparison tools has changed the landscape, and facilitated the wide spread use of both the ligand-based and receptor-based shape-matching technologies in drug discovery (87).

A first example, showing that some of the challenges concerning peptide-based drugs can be successfully overcome, is the introduction of the T-20 peptide (Fuzeon), that signalled a new era in the AIDS therapy (88).

In our view considering the global scenario, the research in the field of antimicrobials is quite fragmented and not supported by the adequate funding that would allow major breakthroughs or the thorough development of already identified lead compounds. The number of different infectious diseases is large, but bacterial diseases usually do not manifest in great epidemics, at least in developed countries, and the limited number of endemic cases of every single disease is too low to draw the necessary huge investments by the pharmaceutical industry. This situation is not supposed to be going to change in the close future, without the intervention of public funding.
